# Systematic analysis of the contributions of stochastic voltage gated channels to neuronal noise

**DOI:** 10.3389/fncom.2014.00105

**Published:** 2014-09-04

**Authors:** Cian O'Donnell, Mark C. W. van Rossum

**Affiliations:** ^1^Computational Neurobiology Laboratory, Salk Institute for Biological StudiesLa Jolla, CA, USA; ^2^School of Informatics, Institute for Adaptive and Neural Computation, University of EdinburghEdinburgh, UK

**Keywords:** channel noise, voltage-gated ion channels, Hodgkin–Huxley, spontaneous firing, simulation

## Abstract

Electrical signaling in neurons is mediated by the opening and closing of large numbers of individual ion channels. The ion channels' state transitions are stochastic and introduce fluctuations in the macroscopic current through ion channel populations. This creates an unavoidable source of intrinsic electrical noise for the neuron, leading to fluctuations in the membrane potential and spontaneous spikes. While this effect is well known, the impact of channel noise on single neuron dynamics remains poorly understood. Most results are based on numerical simulations. There is no agreement, even in theoretical studies, on which ion channel type is the dominant noise source, nor how inclusion of additional ion channel types affects voltage noise. Here we describe a framework to calculate voltage noise directly from an arbitrary set of ion channel models, and discuss how this can be use to estimate spontaneous spike rates.

## 1. Introduction

An obvious characteristic of behavior is the variability that one observes from trial to trial in even the most controlled settings. This behavioral variability is reflected at the neural level in the noisy character of spike trains. Various hypotheses have been put forward for a potential functional role of neural variability, such as stochastic resonance (McDonnell and Abbott, 2009), prevention of synchrony (van Rossum et al., 2002), and probabilistic sampling (Buesing et al., 2011). A number of factors can contribute to trial-to-trial variability: non-stationarity and unobserved modulation of the nervous system; chaotic network dynamics resulting from deterministic single neuron dynamics (van Vreeswijk and Sompolinsky, 1996); and biophysical noise. In this paper we concentrate on the latter, and in particular on the noise from voltage-gated ion channels.

Ion channels are pore-forming macromolecular proteins that allow the selective passage of ionic currents in and/or out of the cell (Hille, 2001). Each ion channel can, at any given moment, occupy only one of multiple discrete conformational states; at least one of which is an open/conducting state, and at least one of which is a closed/non-conducting state. Transitions between states are exceedingly rapid (<1 μs) and, like all molecular reactions, stochastic in nature—they are driven by thermal agitation. In the case of voltage-gated channels (VGCs) considered here, the transition probabilities depend on the cell's membrane potential. Channels are commonly modeled as Markov processes, which lead to accurate predictions of the noise in macroscopic currents recorded from neurons (Hille, 2001).

Because spike generation appears reliable during somatic current injection (Calvin and Stevens, 1968; Bryant and Segundo, 1976; Mainen and Sejnowski, 1995) and the number of VGCs is large, it is typically believed that the stochastic gating of VGC contributes little to the total observed variability in neuronal spiking. However, such a conclusion might be premature. First, during somatic current injection the dendrites are typically more hyper-polarized compared to the realistic case where the neuron receives synaptic input, and the noise typically decreases strongly with hyper-polarization (see below). Moreover, the total number of channels in a neuron might be large, but in spatial compartments such as narrow axons or dendrites, the number of channels is typically small.

Several experimental studies have focused on the physiological consequences of ion channel noise. Sigworth (1980) used fluctuation analysis to estimate the number of Na^+^ channels at a single frog node of Ranvier ~30000, and subsequently used formulae from Lecar and Nossal (1971a,b) to estimate fluctuations in the current threshold of action potential generation due to channel noise. Johansson and Arhem (1994) found that the stochastic opening of a small number of channels in cultured hippocampal neurons were sufficient to trigger spontaneous action potentials. White et al. (1998) recorded subthreshold membrane potential oscillations in stellate cells of layer II entorhinal cortex (EC) and found that they could only reproduce the co-existence of both oscillations and spiking in a computational model if they included stochastic gating of Na^+^ channels, suggesting a form of periodic stochastic resonance. Subsequently, Dorval and White (2005) used the dynamic clamp technique to inject a “virtual” Na^+^ conductance which was either deterministic or stochastic to EC stellate cells *in vitro*. Only the stochastic conductance could reproduce the observed membrane potential oscillations. Similarly, Dudman and Nolan (2009) used computational models of the same cell type to demonstrate that stochastic channel gating can also account for the clustered firing patterns displayed by these cells when stimulated by steady current pulses *in vitro*. Diba et al. (2004) characterized somatic subthreshold voltage noise in cultured hippocampal neurons due to stochastic ion channel gating. Voltage fluctuations were small, with a standard deviation <0.3 mV and based on pharmacological experiments appeared to arise primarily from K^+^ channels. Jacobson et al. (2005) reported similar results from neocortical pyramidal cells from layer IV/V of rat somatosensory cortex brain slices, with similar amplitude (submillivolt) voltage fluctuations. Finally, Kole et al. (2006) used fluctuation analysis to measure the properties and distribution of hyperpolarization-activated cation (Ih) channels in LV neocortical pyramidal cells *in vitro*. They found that although the Ih single-channel conductance was exceedingly small (<1 pS), Ih channels contribute substantially to voltage noise in the distal dendrites of these cells.

A great deal of theoretical and numerical studies have looked at membrane noise from stochastic ion channels, beginning with Lecar and Nossal, who used stochastic differential equations and a reduced dynamical system model of the action potential to attempt to quantify the magnitude of membrane noise on action potential threshold fluctuations (Lecar and Nossal, 1971a,b). Skaugen and Walløe (1979) were the first to examine the consequences of stochastic gating of ion channels through numerical simulation. They found that in the stochastic version of Hodgkin–Huxley (HH) squid giant axon model the current threshold was lowered compared to deterministic models, the membrane could spike spontaneously, and that the frequency-current curve was smeared around the threshold. Subsequent simulation work by DeFelice and colleagues (Clay and DeFelice, 1983; Strassberg and DeFelice, 1993) further elaborated on the direct link between the microscopic (stochastic) and macroscopic (deterministic) versions of the HH model. Rubinstein (1995) simulated a model of the frog node of Ranvier and reproduced the spread in action potential firing threshold due to stochastic channel gating predicted by Lecar and Nossal, in agreement with earlier experiments (Verveen, 1960). Chow and White (1996) examined the dependence of spontaneous firing rate in the stochastic HH model on membrane patch area and found it to decrease exponentially with area. They approximated the system as a boundary escape problem, with stochastic gating of the activation subunit of the Na^+^ channel as the noise source. They found that the mean escape time as a function of area agreed well with numerical simulation results (we will comment on this finding below). Manwani and Koch (1999) used a perturbative approach to compare the contributions of thermal noise, channel noise and synaptic “noise” (from Poissonian inputs) to total membrane voltage noise in a single compartment. Steinmetz et al. (2000) used similar methods to demonstrate the voltage and channel type dependence of ion channel noise spectra for both the HH model and a commonly used neocortical pyramidal cell model (Mainen et al., 1995). In the present study we employ methods very similar to both of these works, but toward a different goal: we aim to systematically separate all of the contributing factors that determine the contribution of an ion channel type to voltage noise and spontaneous firing.

In general detailed simulation of stochastic channels will give the most accurate answer regarding the noise and the contribution of the different channels. But as stochastic simulation of the full channel kinetics is very involved, several recent studies have developed approximate stochastic-differential equation models that efficiently capture the essence of the noise statistics of discrete ion channels (Goldwyn and Shea-Brown, 2011; Goldwyn et al., 2011; Linaro et al., 2011; Orio and Soudry, 2012). Our objective here is complementary but different: rather than developing a precise model for the noise we seek to *estimate* the contribution of the various channel types. Intuitively it is not clear what properties of a given channel type are important determinants for noise. This is relevant when a full state diagram of a certain channel type is not available, but nevertheless a coarse estimate of its contribution to noise is desired. At the same time by breaking down the various factors that determine the magnitude of the noise of a certain channel type, a deeper insight in the results from simulations and experiments can be obtained.

Our study is split in four parts: We use simulations to demonstrate that in the well-studied stochastic Hodgkin–Huxley model, most spikes are due to stochastic K^+^ channel gating and not the Na^+^ channel. This is of interest as in the literature conflicting findings can be found (see Discussion). Next, we review the different factors that explain how stochastic channel noise leads to noise in membrane voltage, using a linear, weak noise analysis and explain why the K^+^ noise is dominant. While these individual contributing factors are well-known, a concise account was in our opinion lacking. Third, we examine the relation between voltage noise and spontaneous spikes using an approach recently introduced for integrate-and-fire neurons. We show that this relation is complex, but that nevertheless rough estimates are possible. Finally, we apply the same methods to analyze a CA1 pyramidal neuron model to show that the approach is easily generalizable to other neuron models.

## 2. Materials and methods

All stochastic channel simulations were implemented using the Parallel Stochastic Ion Channel Simulator (PSICS) which is specifically designed for efficient simulations of stochastic ion channel gating in single neuron models (see Cannon et al., 2010 and http://psics.org/ for algorithmic details). Current noise injection in deterministic HH models was done using NEURON (Carnevale and Hines, 2006). Analysis was done using MATLAB (The Mathworks).

### 2.1. Hodgkin–huxley models

All simulations of the Hodgkin–Huxley model used the standard voltage-dependent equations for Na^+^ and K^+^ gating schemes, at 6.3°C (Hodgkin and Huxley, 1952). For completeness the standard parameters are given in Table 1.

**Table 1:** **The Hodgkin–Huxley parameters for model simulations**.

*C_m_*	Membrane capacitance	1 μF/cm^2^
γ_*Na*_	Na^+^ single-channel conductance	20 pS
ρ_*Na*_	Na^+^ channel density	60 /μm^2^
*E_Na_*	Na^+^ reversal potential	+50 mV
γ_*K*_	K^+^ single-channel conductance	20 pS
ρ_*K*_	K^+^ channel density	18/μm^2^
*E_K_*	K^+^ reversal potential	−77 mV
ρ_*Leak*_	Leak conductance density	3 pS/μm^2^
*E_Leak_*	Leak reversal potential	−55 mV
*V_m_*	Resting membrane potential	−65 mV

In the HH squid axon model, the sodium conductance obeys

$$g_{Na}(V,t)=\gamma_{Na}\rho_{Na}Am^{3}(V,t)h(V,t)$$

where γ_*Na*_ is the single channel sodium conductance, ρ_*Na*_ is the density of channels per area, and *A* is the area. The gating variables *m* and *h* move between off and on-states with a voltage-dependent rate α_*m, h*_(*V*) from the off-state to the on-state, and back with a rate β_*m, h*_(*V*). These rates have been empirically established as

$$\begin{array}{l} \alpha_{m}(V)=\frac{0.1(V+40)}{1-e^{-(V+40)/10}}\;\beta_{m}(V)=4e^{-(V+65)/18}\\ \alpha_{h}(V)=0.07e^{-(V+65)/20}\;\beta_{h}(V)=\frac{1}{1+e^{-(V+35)/10}} \end{array}$$

where *V* is membrane voltage in mV and the transition rates have units 1/ms.

In the limit of very many channels the gating variables are a continuous quantity, namely the probability to find them in the on-state. The dynamics of gating variable *m*(*V,t*) can be written as

(1) $$\frac{dm(V,t)}{dt}=\frac{m_{\infty }(V)-m(V,t)}{\tau_{m}(V)}$$

where *m*_∞_(*V*) is the steady-state value for the activation variable,

(2) $$m_{\infty }(V)=\frac{\alpha_{m}(V)}{\alpha_{m}(V)+\beta_{m}(V)}$$

and τ_*m*_(*V*) is its time constant

(3) $$\tau_{m}(V)=\frac{1}{\alpha_{m}(V)+\beta_{m}(V)}$$

and analogous for the inactivation variable *h*(*V,t*). The dynamics in the continuum limit is fully deterministic.

When the system is described stochastically, the gating variables of each channel are binary variables that switch between off-state and on-state. For the Na^+^ channel to be open, all its 3 *m*'s and the *h* switch need to be in the on-state. To (naively) simulate this case the transitions are drawn stochastically using a random number generator, using a time-step δ*t* such that αδ*t*, βδ*t* ≪ 1.

Likewise, the K^+^ conductance is given by

$$g_{K}(V,t)=\gamma_{K}\rho_{K}An^{4}(V,t)$$

It has four identical activation variables, labeled *n*, with rates

$$\alpha_{n}(V)=\frac{0.01(V+55)}{1-e^{-(V+55)/10}}\;\beta_{n}(V)=0.125e^{-(V+65)/80}$$

In all simulations the single channel conductance for both Na^+^ and K^+^ was 20 pS. Although this value is close to that reported experimentally for the squid giant axon K^+^ conductance (Llano et al., 1988), it is slightly larger than experimental estimates for the Na^+^ conductance (Bezanilla, 1987). These values were chosen for simplicity (it removes one confounding factor when comparing channel type noise contributions) and to enable comparison with the literature (Strassberg and DeFelice, 1993; Chow and White, 1996; Schneidman et al., 1998). In line with the literature, leak channels were modeled deterministically, although in more realistic models they should be made stochastic as well.

For the simulations and analysis of the hippocampal pyramidal cell model (**Figure 6**), we use the channel models for active Na^+^, delayed rectifier K^+^ (Kdr), and A-type K^+^ channel (Ka) exactly as previously published by Migliore et al. (1999), Jarsky et al. (2005). However, our model was single-compartment while these previous studies looked at multi-compartment model neurons. For consistency with the HH simulations we also choose a single-channel conductance of 20 pS. The channel densities were matched to the macroscopic conductance densities of the soma in the model of Jarsky et al. (2005), implying Na^+^: 20 channels /μm^2^, Kdr: 20/μm^2^, and Ka: 24/μm^2^. In addition to these active channels, we added two voltage-independent leak channels, one permeable to Na^+^ and one permeable to K^+^, which we simulated deterministically. We chose their densities 0.0065 mS/cm^2^ (Na^+^) and 0.0185 mS/cm^2^ (K^+^), to fit two constraints: a total leak conductance of 0.025 mS/cm^2^ (Jarsky et al., 2005), and a resting voltage of −65 mV. Finally, as according to Migliore et al. (1999) we set reversal potentials *E*_*Na*_ = +55 mV and *E_K_* = −90 mV. When attempting to analytically calculate the membrane impedance for this model we unfortunately found that it diverged upward at around −60 mV. This singularity is problematic because it would break our small voltage noise assumption. Hence we instead estimated the impedance for this model empirically as is done in experiments. We injected sine wave currents over a large frequency range to the deterministic version of the model, and measured the amplitude of the resulting voltage responses.

### 2.2. Calculation of powerspectra of K^+^ and Na^+^ noise

As is well known, the current noise power spectrum from a population of ion channels follows directly from the channel kinetic scheme and its autocovariance function (DeFelice, 1981). For ease of presentation we first summarize this calculation for a two-state channel. The conditional probability that a two-state channel is open at time *t* given that it was open at time 0, *p*_*o*|*o*_(*t*) is

$$p_{o|o}(t)=p_{\infty }+(1-p_{\infty })e^{-t/\tau }$$

where *p*_∞_ is the steady-state open probability and τ is the correlation time. The autocorrelation 〈*p_o_*(*t*_0_)*p_o_*(*t*) 〉 = *p*_∞_*p*_*o*|*o*_(*t*). The autocovariance of a single channel *C_o_*(*t*) open probability can then be written as

$$\begin{array}{l} C_{o}(t)=\langle \left[p_{o}(t_{0})-p_{\infty }\right]\left[p_{o}(t)-p_{\infty }\right]\rangle \\ \;=p_{\infty }p_{o|o}(t)-p_{\infty }^{2}\\ \;=p_{\infty }(1-p_{\infty })e^{-t/\tau } \end{array}$$

The autocovariance of the current through a population of *N* such channels, *C_I_*(*t*), is simply given by

$$C_{I}(t)=Ni^{2}C_{o}(t)$$

where *i* is the single-channel current. Note that the autocovariance function at *t* = 0 is equal to the variance, *C_I_*(0) = *Ni*^2^*p*_∞_ (1 − *p*_∞_) = σ^2^, and decays exponentially with time constant τ, so that when *t* ≫ τ, *C_I_*(*t*) → 0.

The Wiener-Khinchin theorem states that the power spectrum is equal to the real part of the Fourier transform of the autocovariance function

(4) $$\begin{array}{l} S_{I}(\omega )=4\int_{0}^{\infty }C_{I}(t)e^{-2\pi ift}dt\\ \;=S_{I}(0)\frac{1}{1+{\left(2\pi f\tau \right)}^{2}} \end{array}$$

where *S_I_*(0) = 4*Ni*^2^*p*_∞_(1 − *p*_∞_)τ and the pre-factor 4 arises from our definition of spectral density. Thus for the two-state channel population, the power spectrum is a single Lorenztian function with a corner frequency *f_c_* = 1/(2π τ). Above this corner frequency, the power of the signal falls off ∝ 1/*f*^2^.

In an analogous way we can calculate the power spectra of the HH Na^+^ and K^+^ channel populations. For the HH K^+^ channel composed of four identical and independent sub-units, the conditional probability that the channel is open at time *t* given that it was open at time 0 is

(5) $$p_{K,o|o}(t)={\left(n_{\infty }+(1-n_{\infty })e^{-t/\tau_{n}}\right)}^{4}$$

Hence, the autocovariance of the current noise from the HH K^+^ channel population is a sum of exponentials,

$$\begin{array}{l} C_{IK}(t)=N_{K}i_{K}^{2}\left(n_{\infty }^{4}p_{K,o|o}(t)-{\left(n_{\infty }^{4}\right)}^{2}\right)\\ \;=N_{K}i_{K}^{2}n_{\infty }^{4}[{\left(1-n_{\infty }\right)}^{4}e^{-4t/\tau_{n}}+4n_{\infty }{\left(1-n_{\infty }\right)}^{3}e^{-3t/\tau_{n}}\\ \;+6n_{\infty }^{2}{\left(1-n_{\infty }\right)}^{2}e^{-2t/\tau_{n}}+4n_{\infty }^{3}(1-n_{\infty })e^{-t/\tau_{n}}] \end{array}$$

The corresponding power spectrum of the current noise is

$$\begin{array}{l} S_{IK}(\omega )=4N_{k}n_{\infty }^{4}i_{K}^{2}\tau_{n}[{\left(1-n_{\infty }\right)}^{4}\frac{4}{16+\omega^{2}\tau_{n}^{2}}\\ \;+n_{\infty }{\left(1-n_{\infty }\right)}^{3}\frac{12}{9+\omega^{2}\tau_{n}^{2}}+n_{\infty }^{2}{\left(1-n_{\infty }\right)}^{2}\frac{12}{4+\omega^{2}\tau_{n}^{2}}\\ \;+n_{\infty }^{3}(1-n_{\infty })\frac{4}{1+\omega^{2}\tau_{n}^{2}}] \end{array}$$

This is the sum of four Lorenztians with corner frequencies equal to 4/(2π τ_*n*_), 3/(2π τ_*n*_), 2/(2π τ_*n*_), and 1/(2π τ_*n*_). Because at the resting potential *n*_∞_ is close to zero, the first term in the square brackets with correlation time τ_*n*_/4 will dominate the power spectrum.

Similarly, for the Na^+^ current noise power spectrum one has

$$p_{Na,o|o}={\left(m_{\infty }+(1-m_{\infty })e^{-t/\tau_{m}}\right)}^{3}\left(h_{\infty }+(1-h_{\infty })e^{-t/\tau_{h}}\right)$$

and the Na^+^ current noise autocovariance is

$$\begin{array}{l} C_{INa}(t)=N_{Na}i_{Na}^{2}\left(m_{\infty }^{3}h_{\infty }p_{Na,o|o}-{\left(m_{\infty }^{3}h_{\infty }\right)}^{2}\right)\\ \;=N_{Na}i_{Na}^{2}m_{\infty }^{3}h_{\infty }[3m_{\infty }^{2}h_{\infty }\left(1-m_{\infty }\right)e^{-t/\tau_{m}}\\ \;+3m_{\infty }h_{\infty }{\left(1-m_{\infty }\right)}^{2}e^{-2t/\tau_{m}}\\ \;+h_{\infty }{\left(1-m_{\infty }\right)}^{3}e^{-3t/\tau_{m}}\\ \;+m_{\infty }^{3}\left(1-h_{\infty }\right)e^{-t/\tau_{h}}\\ \;+3m_{\infty }^{2}(1-m_{\infty })(1-h_{\infty })e^{-t/\tau_{m}-t/\tau_{h}}\\ \;+3m_{\infty }{\left(1-m_{\infty }\right)}^{2}\left(1-h_{\infty }\right)e^{-2t/\tau_{m}-t/\tau_{h}}\\ \;+{\left(1-m_{\infty }\right)}^{3}\left(1-h_{\infty }\right)e^{-3t/\tau_{m}-t/\tau_{h}}] \end{array}$$

The corresponding power spectrum of the Na^+^ current noise is

$$\begin{array}{l} S_{INa}(\omega )=4N_{Na}i_{Na}^{2}{\left(m_{\infty }^{3}h_{\infty }\right)}^{2}[\left(\frac{1-m_{\infty }}{m_{\infty }}\right)\frac{3\tau_{m}}{1+\omega^{2}\tau_{m}^{2}}\\ \;+{\left(\frac{1-m_{\infty }}{m_{\infty }}\right)}^{2}\frac{6\tau_{m}}{4+\omega^{2}\tau_{m}^{2}}+{\left(\frac{1-m_{\infty }}{m_{\infty }}\right)}^{3}\frac{3\tau_{m}}{9+\omega^{2}\tau_{m}^{2}}\\ \;+\left(\frac{1-h_{\infty }}{h_{\infty }}\right)\frac{\tau_{h}}{1+\omega^{2}\tau_{h}^{2}}\\ \;+3\left(\frac{1-h_{\infty }}{h_{\infty }}\right)\left(\frac{1-m_{\infty }}{m_{\infty }}\right)\left(\frac{\tau_{m}\tau_{h}}{\tau_{m}+\tau_{h}}\right)\\ \;\frac{1}{1+{\left(\omega \tau_{m}\tau_{h}/(\tau_{m}+\tau_{h})\right)}^{2}}\\ \;+3\left(\frac{1-h_{\infty }}{h_{\infty }}\right){\left(\frac{1-m_{\infty }}{m_{\infty }}\right)}^{2}\left(\frac{\tau_{m}\tau_{h}}{\tau_{m}+2\tau_{h}}\right)\\ \;\frac{1}{1+{\left(\omega \tau_{m}\tau_{h}/(\tau_{m}+2\tau_{h})\right)}^{2}}\\ \;+\left(\frac{1-h_{\infty }}{h_{\infty }}\right){\left(\frac{1-m_{\infty }}{m_{\infty }}\right)}^{3}\left(\frac{\tau_{m}\tau_{h}}{\tau_{m}+3\tau_{h}}\right)\\ \;\frac{1}{1+{\left(\omega \tau_{m}\tau_{h}/(\tau_{m}+3\tau_{h})\right)}^{2}}] \end{array}$$

Near rest *m*_∞_ ≪ 1 and *h*_∞_ ≈ 0.6, so that the third and last Lorentzians dominate the powerspectrum. The corresponding corner-frequencies are 3/(2π τ_*m*_) and (τ_*m*_ + 3τ_*h*_)/(2π τ_*m*_τ_*h*_), which are virtually identical.

We calculate the quasi-active (linearized) membrane impedance using standard methods (Mauro et al., 1970; Koch, 1999).

## 3. Results

### 3.1. Stochastic potassium channels can trigger spontaneous action potentials

Previously, it has been demonstrated that a Hodgkin–Huxley (HH) type neural model with discrete Markovian stochastic ion channels instead of the classic continuous deterministic rate equations can fire spontaneous action potentials if the membrane patch is small (Skaugen and Walløe, 1979; Clay and DeFelice, 1983; Strassberg and DeFelice, 1993; Chow and White, 1996; Schneidman et al., 1998). However, the relative contributions of the Na^+^ and K^+^ channel populations to spontaneous activity are less well understood. To investigate, we simulate the HH squid axon model using the PSICS simulator (Cannon et al., 2010) with stochastic Markovian ion channels while varying the membrane patch area under three different conditions: first, both sodium (Na^+^) and potassium (K^+^) channels stochastic (“all stochastic”), second, Na^+^ stochastic but K^+^ deterministic, and third, Na^+^ deterministic but K^+^ stochastic. Comparing the spontaneous firing rate between the three conditions allows us to find whether Na^+^ or K^+^ channels contribute most to spontaneous activity.

**Figure 1:**
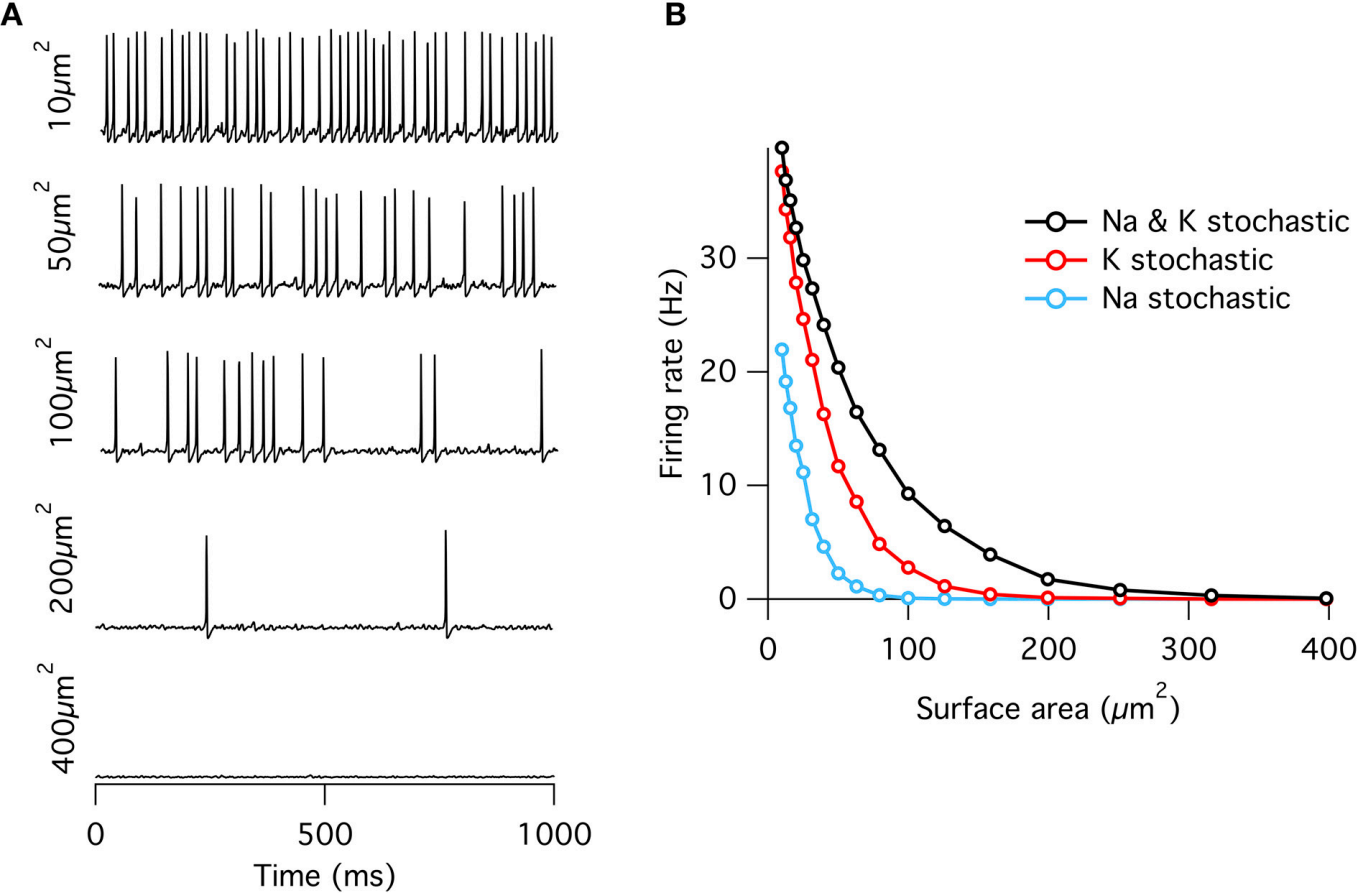
**Spontaneous action potentials in an isopotential Hodgkin–Huxley model**. **(A)** Example membrane potential traces from the single compartment stochastic HH model of varying membrane surface areas. **(B)** Spontaneous firing rate decreases approximately exponentially with increasing surface area. Firing rates at all areas were greater for the “all stochastic” model (black) than the K^+^ stochastic model (red), which was in turn greater than the Na^+^ stochastic model (blue).

As observed previously (Chow and White, 1996), if a fixed density of ion channels is assumed, then the firing rate decreases approximately exponentially with increasing membrane patch area (Figure 1) such that membrane areas greater than ~400 μm^2^ produce almost no spontaneous action potentials approximating the deterministic model. This exponential dependence of spontaneous rate with membrane area is consistent with a stochastic barrier-escape problem (Chow and White, 1996). As the channels are independent, the voltage variance is proportional to the number of channels *N*, but inversely proportional to the square of the area *A*, since the input impedance decreases linearly with area. Combining these two opposing factors, the spontaneous rate scales as exp( − *A*^2^/*N*) ∝ exp( − *A*).

When either Na^+^ or K^+^ channels are switched to deterministic mode, spontaneous firing rate is reduced compared to the fully stochastic mode. Surprisingly, however, stochastic K^+^-channel gating alone triggers greater spontaneous firing rates than stochastic Na^+^-channel gating alone (Figure 1B). At first impression, this result might be counter-intuitive because the opening of Na^+^ channels is necessary for the initiation of an action potential, while the much slower K^+^ channels are conventionally considered responsible for the re-polarizing phase. A simple conceptual model for spontaneous spike generation might therefore be that the chance opening of a few Na^+^ channels depolarizes the membrane and activates the runaway Na^+^ channel opening underlying the action potential. However, stochastic *closure* of K^+^ channels can also depolarize the membrane, similarly activating Na^+^ channels to trigger an action potential. We test this possibility by examining the dynamics of Na^+^ and K^+^ currents. We adapt the ‘spike-triggered average’ (STA) measure from the neural coding literature. Here we determine the average total current of a given ion channel population *x* at time interval *t* prior to a spontaneous action potential at time *t_i_*, averaged over *n* such events,

$$I_{x}^{STA}(t)=\frac{1}{n}\left[\sum_{i=1}^{n}I_{x}(t_{i}-t)\right]$$

**Figure 2:**
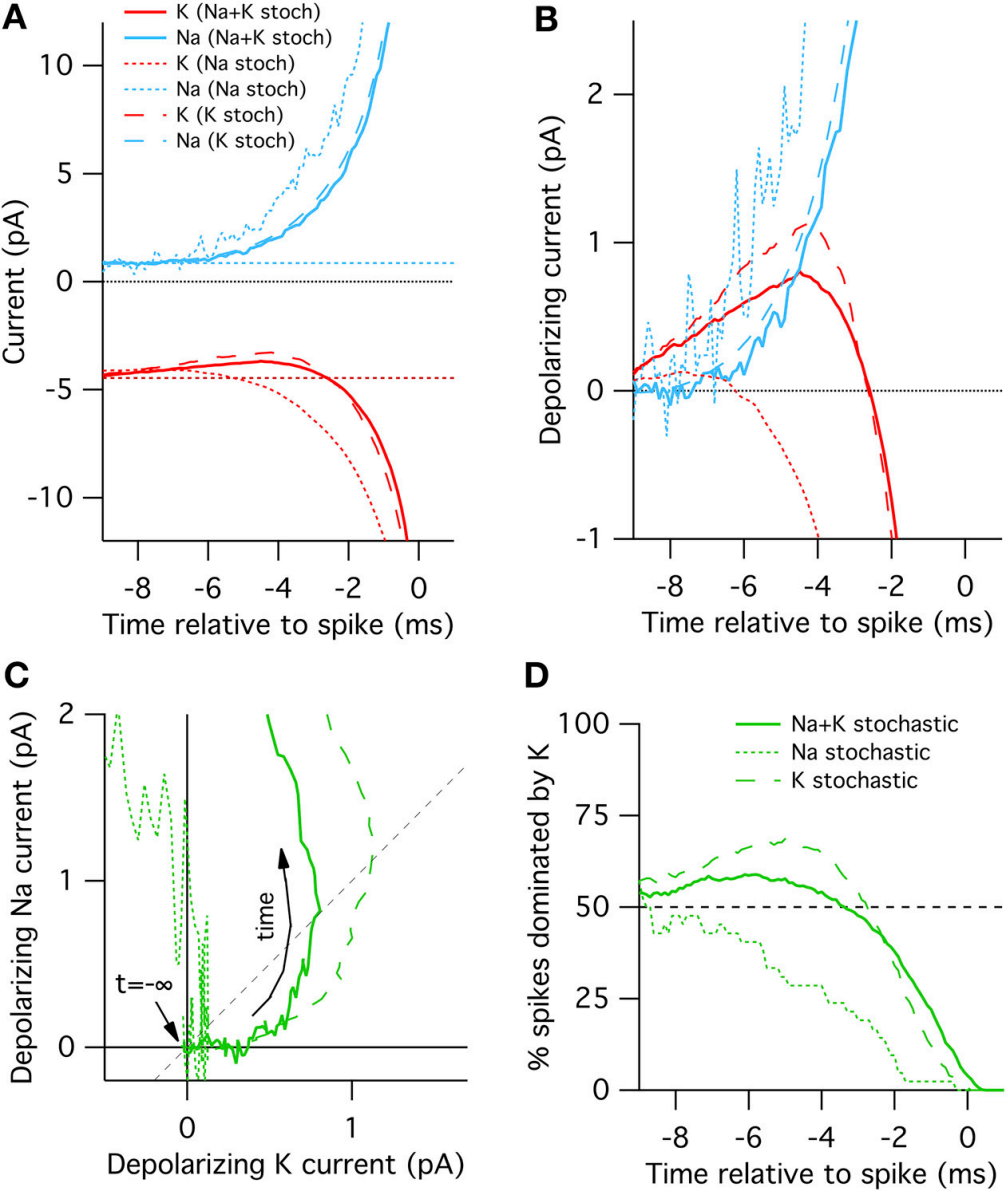
**Spike-triggered averaged Na^+^ and K^+^ currents preceding a spontaneous action potential in a 100 μm^2^ surface area model neuron**. **(A)** STA currents from Na^+^ (blue) and K^+^ (red) channels. Curves shown for three conditions: all channels stochastic (solid), Na^+^ only stochastic (dotted) and K^+^ only stochastic (dashed). In line with convention, depolarizing currents are plotted negative. **(B)** Average change in Na^+^ and K^+^ currents compared to rest in time preceding spontaneous action potential. **(C)** Average change Na currents vs. K currents during spontaneous spikes. Same data as in **(B)**. Dashed diagonal line denotes identity where Δ*I_Na_*(*t*) = Δ*I_K_*(*t*). **(D)** Proportion of trials where the K current exceeds the Na current, Δ*I_K_*(*t*) > Δ*I_Na_*(*t*).

In the “all stochastic” mode (Figure 2A, solid curves), we find that the STA potassium current *I*^*STA*^_*K*_(*t*), drops between 8 and 2 ms before the spike, while there is a simultaneous increase in the STA sodium current *I*^*STA*^_*Na*_(*t*). Nearer to the spike the Na^+^ and K^+^ currents grow rapidly but in opposite directions as the action potential forms. A positive current corresponds to depolarization. This depolarizing action of the K^+^ current change be clearly seen in Figure 2B where we plot Δ*I_K_*(*t*) and Δ*I_Na_*(*t*), the change in Na^+^ and K^+^ relative to resting current. Importantly, the change in *I*^*STA*^_*K*_ precedes the increase in *I*^*STA*^_*Na*_ (Figure 2B), suggesting that spontaneous action potential firing in this model is primarily driven by K^+^ channel fluctuations, not Na^+^ noise. We test this explanation by simulating K^+^ channel conductance in deterministic mode and repeating the STA measurement. As expected, in this case spontaneous spikes are not preceded by a drop in K^+^ conductance, but instead driven by an elevated Na^+^ conductance fluctuation (Figure 2A, dotted curves).

Further examination of the Na^+^ and K^+^ current dynamics confirm these findings. In Figure 2C we plot the timecourse of Δ*I_Na_*(*t*) (y-axis) vs. Δ*I_K_*(*t*) (x-axis) from 10 to 1 ms prior to each recorded action potential together on the same plot. Regions to the lower-right of the identity diagonal (dashed black line) indicate timepoints where K^+^ current fluctuations are contributing more to voltage depolarization than Na^+^ current fluctuations. The mean STA curve for the “all stochastic” model (solid curve) initially takes off into this right-hand region. In contrast, the Na^+^-only stochastic (dotted curve) moves only slightly to the right of the origin before taking off in the vertical (Na^+^-driven) direction.

Figures 2A–C plot the average behavior. As the system is stochastic, we expect that occasionally Na^+^ fluctuations trigger spikes as well. To get a sense of the spike-to-spike variability in Figure 2D we plot the percentage of cases where the potassium current exceeds the sodium current, Δ*I_K_*(*t*)> Δ*I_Na_*(*t*), as a function of time before spike. In general, this quantity is time-dependent because the depolarization due to K^+^ fluctuations will most likely be maximal at some time between 8 and 2 ms before the spike. Once the full spike upswing begins (~1 ms before *t* = 0) Na^+^ always dominates. In the all-stochastic case, the earliest phase of most action potentials are K^+^-driven. In contrast, in the Na^+^ stochastic case (dotted line) the majority of spikes are driven primarily by Na^+^ fluctuations.

A slightly different picture appears for the K^+^ stochastic simulations. In this mode, Na^+^ fluctuations are removed so all spikes must by initially triggered by K^+^ fluctuations. Consequently, the spike-triggered average K^+^ current fluctuation amplitude is even greater than in the all-stochastic model (Figures 2A–C) and an even larger percentage of spikes are driven by momentary fluctuations in K^+^ currents (Figure 2D). In summary, these simulations show that K^+^ channel noise is the dominant driver of spontaneous spiking in the stochastic Hodgkin–Huxley model.

Schneidman et al. (1998) looked at stochastic Na^+^ and K^+^ channel trajectories during spike initiation to address whether a sufficiently strong stimulus can override the intrinsic channel noise. However, in contrast to our STA analysis for determining the contributions of Na^+^ vs. K^+^ noise to spontaneous spiking, they examined stimulus-driven firing by injecting a fluctuating current into the model neuron. In this stimulus-driven case, the observed trajectories of the Na^+^ vs. K^+^ currents combine the effect of the stimulus current dynamics and the effect of the channel noise. Our analysis however shows that the drop in noisy K^+^ current occurs naturally before the spike.

### 3.2. The factors determining a conductance's contribution to membrane noise

These results lead to the questions: What properties of the HH K^+^ conductance cause it to trigger more spontaneous action potentials than the Na^+^ conductance? And how can the contribution of an arbitrary channel type be estimated? We proceed by first calculating the resulting noise in the membrane voltage. In the limit of large areas (small noise), this can be calculated exactly. In the subsequent section we relate the voltage noise to spontaneous spike rates. This will turn out to be only approximately possible.

There are at least five possible factors that determine a channel population's contribution to membrane noise:

#### 3.2.1. Open probability (11 × noisier for K^+^)

First, Na^+^ and K^+^ have different steady-state open probabilities at resting membrane potential. The steady-state probability of a single ion channel being open is identical to the steady-state permeability fraction of the corresponding macroscopic conductance in the classic HH formalism. The steady-state Na^+^ conductance is obtained from the product of the steady-state values of the *m*_∞_ and *h*_∞_ gating variables:

$$g_{Na\infty }(V)={\bar{g}}_{Na}{\left[m_{\infty }(V)\right]}^{3}h_{\infty }(V)$$

where *g*_*Na*_ is the maximal conductance through the Na^+^ channel population. Hence the open probability *p*_o_ = (*m*_∞_)^3^*h*_∞_. The gating variables *m*_∞_ and *h*_∞_ can in turn be expressed in terms of the forward and backward gating rates α_*m*_ and β_*m*_, and α_*h*_ and β_*h*_, see Equation 2. The steady-state K^+^ open probability equals *p_o_* = [*n*_∞_(*V*)]^4^. At the resting potential of −65 mV in the HH squid axon model, the steady-state open probabilities are ~0.000089 for the Na^+^ and and ~0.010 for the K^+^ channels. At any instant the open probability follows a binomial distribution so that the variance in the single channel current σ^2^_*i*_ = *i*^2^*p_o_*(1 − *p_o_*), where *i* is the single-channel current. The variance is parabolic in *p_o_*: zero when *p_o_* = 0 or 1, and maximal at *p_o_* = 0.5. As below spike threshold, most ion channels have very low open probabilities, the standard deviation can be approximated by

$$\sigma_{i}=i\sqrt{p_{o}}$$

Therefore, ion channel populations with greater *p_o_* at resting membrane potential tend to have larger current fluctuations than populations with lesser *p_o_*. This effect predicts that the standard deviation of the K^+^ channel noise is 10.7 times larger than Na^+^ channel noise.

#### 3.2.2. Number of channels (1.8 × noisier for Na^+^)

Second, because the channels act independently, the standard deviation of the number of open channels grows proportional to $\sqrt{N}$. Thus channel populations with greater *N* have greater fluctuations in their absolute number of open channels. The Na^+^ population has 3.33× more channels in the standard HH model than the K^+^ population (Table 1), yielding Na standard deviation larger by a factor $\sqrt{10/3}\approx 1.8$.

#### 3.2.3. Driving force (10 × noisier for Na^+^)

The third factor is the difference in driving force for each conductance. As the HH model assumes that these ion channel current-conductance relationships are Ohmic (linear), the current through an open channel is proportional to the difference between the membrane potential and the channel's driving force,

$$i_{x}=\gamma_{x}(V_{m}-E_{x})$$

where *i_x_* is the single-channel current, γ_*x*_ is the single-channel conductance, *V_m_* is the membrane potential and *E_x_* is the conductance's reversal potential, given by the Nernst equation. In the HH model, *E_Na_* = +50 mV, *E_K_* = −77 mV, and *V_rest_* = −65 mV, giving Na^+^ a driving force of +115 mV and K^+^ a driving force of −12 mV. This means that the driving force for the Na^+^ current is 9.6× greater than the K^+^ current at *V_rest_*.

#### 3.2.4. Single-channel conductance (identical for Na^+^ and K^+^)

Fourth, the single-channel conductance γ_*x*_ is another important factor determining a channel's contribution to membrane noise. For the same channel population current per unit squared cell membrane, a larger γ_*x*_ implies smaller *N*, and a larger *i*_*x*_. Hence channels with a larger single-channel conductance will have greater current fluctuations. In our implementation of the stochastic HH model, however, we assume, like most other stochastic models, the same single-channel conductance for both Na^+^ and K^+^ (20 pS). The value of 20 pS is close to experimentally measured estimates for Na+ (14 pS) (Bezanilla, 1987), while the K^+^ conductance in the squid axon is probably made up of multiple different channel types, with single-channel conductances estimated at 10, 20, and 40 pS (Llano et al., 1988).

**Figure 3:**
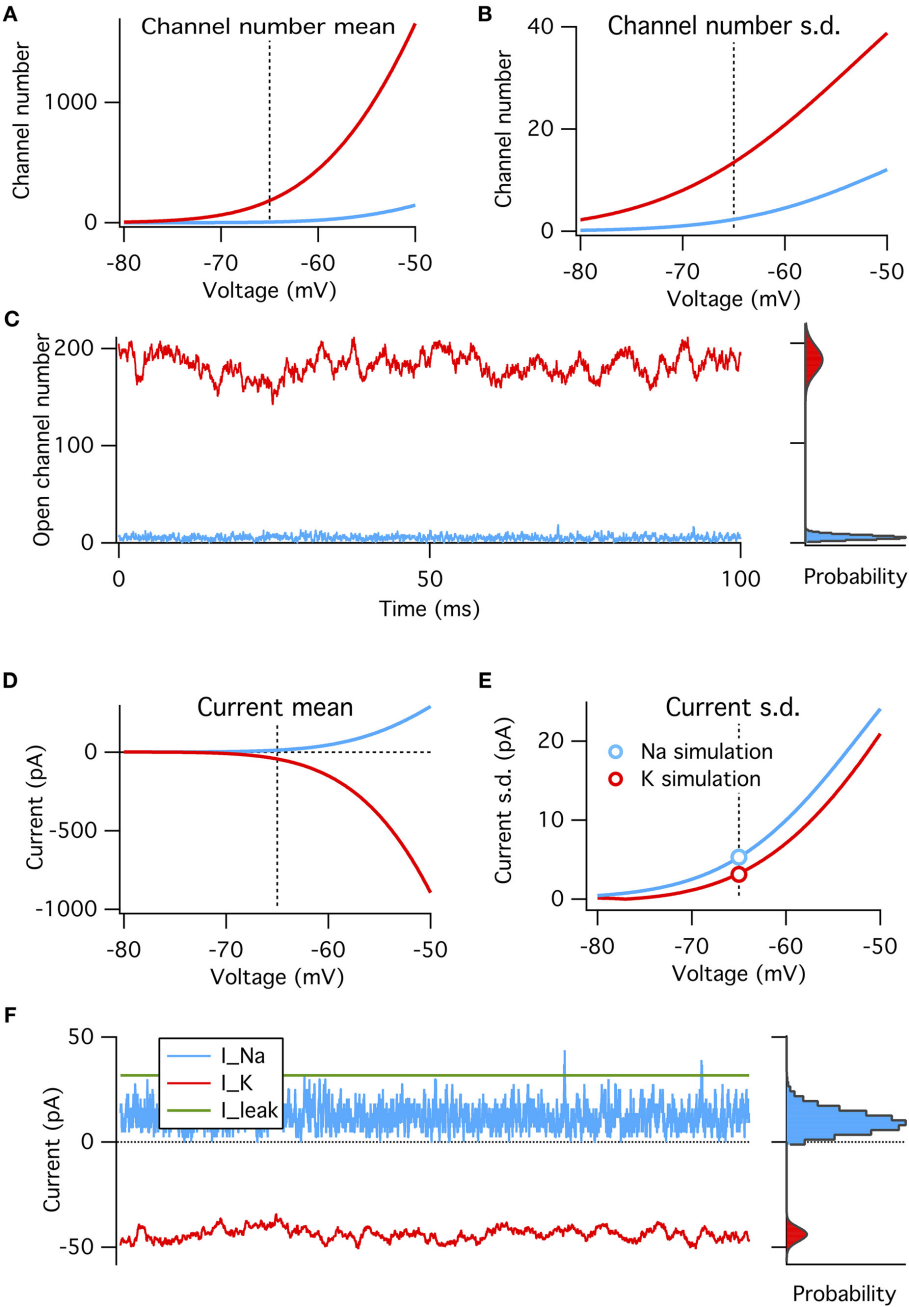
**A binomial model reproduces the steady-state features of simulated voltage clamp data at −65 mV**. **(A)** Mean number of open channels as function of voltage for Na^+^ (blue) and K^+^ (red) HH conductances. Dotted vertical line in all panels indicates resting voltage, −65 mV. **(B)** Variance in number of open channels for conductances in **(A)**. **(C)** Example time series of Na^+^ and K^+^ open channels numbers from voltage-clamp simulation at resting potential (left) with histogram of open channel numbers (right). Gray curves overlaying the right histograms are the binomial prediction. **(D–F)** Similar to **(A–C)** but for total channel population currents instead of open numbers. Modeled for membrane area of 1000 μm^2^.

These four factors can be put together to construct a binomial model of the amplitude of channel noise at steady state. This model does not have any notion of dynamics or channel kinetics. We calculate the steady-state open probabilities at rest directly from the Hodgkin–Huxley equations, and test the binomial model's ability to reproduce simulated voltage-clamp data and probe its predictions on the relative magnitudes of Na^+^ and K^+^ channel noise (Figure 3).

We find that, as expected, the binomial model exactly predicts the conductance and current fluctuations from voltage-clamp simulation data at resting potential of −65 mV (Figures 3C,F). We use the binomial model to estimate the steady-state standard deviation in open channel numbers and total current from the Na^+^ and K^+^ populations at a range of membrane potentials (Figures 3A–B,D–E). As expected from the above analysis the Na^+^ current standard deviation is about 1.7× that of the K^+^ current (matched in simulations, see circle symbols in Figure 3E).

#### 3.2.5. Channel gating kinetics and membrane filtering (3 × noisier for K^+^)

The fifth factor is that the Na^+^ and K^+^ conductances have different gating kinetics. These differences are important because the current fluctuations from each ion channel populations are filtered differentially by the membrane impedance, hence altering each channel's contributions to membrane voltage noise.

**Figure 4:**
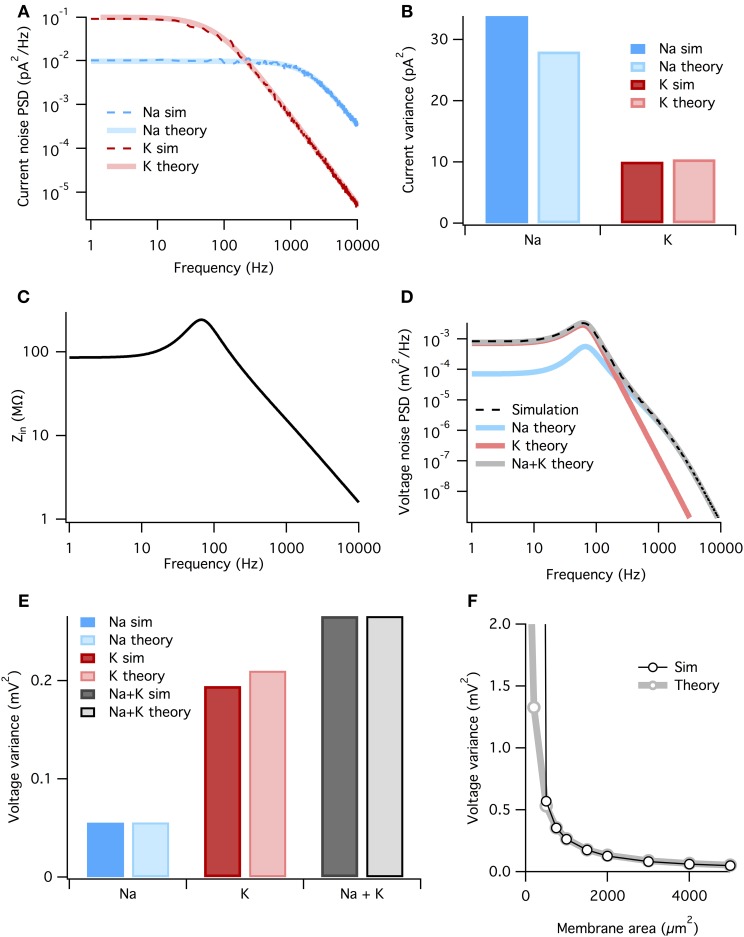
**The membrane filters Na^+^ noise more than K^+^ noise**. **(A)** Spectral density of current noise from HH Na^+^ and K^+^ channels. Thin dashed dark colored curves are PSD estimates from simulation data, thick light colored curves are theoretical, derived from channel kinetic schemes. **(B)** Variance of Na^+^ and K^+^ current noise from both simulation and theory. Note Na^+^ channel noise variance is greater than K^+^ channel noise. **(C)** Total HH membrane impedance at −65 mV as a function of signal frequency. **(D)** Voltage noise of Na^+^ and K^+^ channels calculated from current noise spectra and membrane impedance. Gray curve is sum of Na^+^ and K^+^ noise, while the dashed black curve is an estimate of the voltage noise spectrum measured from simulation data. **(E)** Theoretical variance of voltage noise from Na^+^ and K^+^ channels compared with estimates from simulation. Note K^+^ channels contribute more to voltage noise than Na^+^ channels. **(F)** Comparison of voltage noise variance from theory and simulation as a function of membrane area. Note curves substantially diverge only for areas <500 μm. For those small areas spontaneous spiking occurs and the associated large voltage fluctuations are not part of the theory. All other panels use a membrane area of 1000 μm^2^.

In the Methods we calculate the power-spectra of the Na^+^ and K^+^ current noise assuming a constant membrane potential and small noise. Both powerspectra are sums of multiple Lorentzians,

$$S_{I}(f)=\sum_{k}\frac{a_{k}}{1+{\left(f/f_{c}^{k}\right)}^{2}}$$

where the *f*^*k*^_*c*_ are the corner frequencies of the Lorentzians (the frequency at which the powerspectrum is half of the zero frequency magnitude), and *a*_*k*_ are (voltage-dependent) coefficients. The full expressions are given in the Methods, but the K^+^ noise spectrum is dominated by a Lorentzian with corner frequency *f_c_* = 4/(2π τ_*n*_). At the resting potential τ_*n*_ ~ 5.5 ms (Equation 3), so that the dominant Lorentzian has a corner frequency of 115 Hz. The Na^+^ spectrum is dominated by a Lorentzian with *f_c_* = 3/(2π τ_*m*_). At rest τ_*m*_ ~ 0.24 ms, τ_*h*_ ~ 8.5 ms, so that the dominant Lorentzian for Na^+^ has a much higher corner frequency of 1980 Hz—note however that we include all Lorentzian terms in the presented results, not just the dominant one. The analytically calculated spectra match well the estimated spectra of the simulated stochastic currents, Figure 4A.

To calculate the voltage response to the current noise, we approximate the active membrane by a linear impedance. The voltage noise power spectrum *S_V_*(*f*) is given by the generalized Ohm's law

$$S_{V}(f)=S_{I}(f)|Z(f){|}^{2}$$

where *S_I_*(*f*) is the power spectrum of a current noise source and *Z*(*f*) is the membrane impedance. The impedance relates changes in voltage to changes in currents. In active membranes the input impedance is not given by just the capacitance and leak conductance, but also by any other channels open at rest and their reaction to small changes in the voltage. The impedance can be found by linearizing the four-dimensional (*V*, *m*, *h*, and *n*) HH equations around the resting state (e.g., Mauro et al., 1970; Koch, 1984; Carnevale and Hines, 2006). In the HH model the presence of Na^+^ and K^+^ conductances introduce a resonance in the impedance at ~100 Hz, but the 1/*f* behavior still dominates at higher frequencies (Figure 4C). The resonant peak in the impedance is the electrical signature of an inductor. Although such an inductor has no physical counterpart in the biological cell membrane, the delayed-rectifier K^+^ conductance opposes changes in membrane potential and for small currents behaves as a phenomenological inductance (Mauro et al., 1970; Koch, 1984, 1999).

Now we combine the membrane impedance with the current noise spectra to calculate each channel population's contribution to voltage noise. In Figure 4D we plot the theoretical power spectra of the voltage noise from the HH Na^+^ and K^+^ channel populations, calculated at *V_m_* = *V_rest_* = −65 mV. The sum of the Na^+^ and K^+^ power spectra give the total voltage noise power spectrum (gray line in Figure 4D). This predicts almost exactly the power spectrum measured from simulation (dashed line in Figure 4D). The voltage noise variance from each channel population equals the integrated power spectrum:

$$\sigma_{x}^{2}=\int_{0}^{\infty }S_{x}(f)df$$

where subscript *x* indicates the relevant channel population. These are graphed in Figure 4E. It is clear the K^+^ channel fluctuations contributes ~ 4× more *voltage* noise variance than Na^+^. While the Na^+^ *current* noise has a greater variance than the K^+^ current noise, it is filtered more strongly by the membrane impedance.

Can we summarize the total effects of membrane filtering on noise from the two channel types? One way to quantify this effect is to take the ratio of voltage and current noise standard deviations for each channel type, *r_x_* = σ_*Vx*_/σ_*Ix*_. Doing so we find that *r_Na_* = 44.5 MΩ and *r_K_* = 141.7 MΩ, implying that, after all other factors are accounted for, membrane filtering attenuates Na^+^ noise ~ 3× more than K^+^ noise.

As both the noise spectra and the impedance are voltage-dependent, these calculations assume that the voltage remains at a fixed potential, which holds if the fluctuations are small. Because here we simulate a large membrane area (1000 μm^2^) with low membrane resistance, voltage changes are small and there is only a small discrepancy between the voltage noise calculated analytically and the estimate from simulation (Figure 4E). In particular for small membrane areas the voltage fluctuations will be large, nevertheless the approximation remains good down until areas where spontaneous spikes appear, Figure 4F. At this point, currents associated to the spike will dominate the measured current.

In summary, the contribution of each channel type to membrane noise is determined by their number, single-channel conductance, voltage dependencies and gating kinetics. This is true for any neuron model. In the case of the HH model, the sum properties of K^+^ channels at subthreshold voltages make their contributions to membrane noise greater than that from Na^+^ channels.

### 3.3. Effect on spontaneous spike rates

So far we have seen that the channels in the HH model contribute differentially to the noise in the membrane voltage and that K^+^ channels have the largest contribution. One would expect that therefore K^+^ channels are the most important contributor of noise-driven spontaneous spike activity as well. This is indeed the case as we have seen in Figure 1 but quantitatively the link between the spontaneous rate and the subthreshold membrane voltage fluctuations is not trivial.

The analysis of spontaneous spiking rate in the HH model to correlated noise is a complicated stochastic differential equation problem. Reducing the spiking mechanism to a one dimensional escape problem, Chow and White (1996) derived the spontaneous rate using multiplicative, white (uncorrelated) current noise to approximate the Na^+^ noise. But it is not obvious how such an analysis can be extended to colored (correlated) noise. The time-constants of the K^+^ noise, Na^+^ noise and the membrane are all of similar magnitude (Figure 4), complicating any perturbative expansion. Even in much simpler integrate-and-fire neuron models, the treatment of correlated noise is complicated, resulting in a two-dimensional Fokker-Planck equation that can only be solved in certain limits (Brunel and Sergi, 1998; Moreno-Bote and Parga, 2004; Alijani and Richardson, 2011).

We first examine how the correlation time in the Gaussian noise model affects spontaneous firing rates in a HH neuron. Traditionally, studies have kept the variance of the injected noise fixed while varying the correlation time. However, as shown above, this can lead to widely different voltage fluctuations due to the differential membrane filtering. The idea we examine here is that instead of the current variance, the voltage variance is a better predictor of the spontaneous rate. This was recently shown in exponential integrate-and-fire neurons: the noise driven firing rate was relatively independent of the noise correlation time when the voltage variance was kept constant (Alijani and Richardson, 2011).

In order to research this in the HH model, we simulated a deterministic HH neuron and injected a Gaussian noise current with a correlation time τ and with a correlation function

$$\langle I(T)I(T+t)\rangle =\frac{\sigma_{V}^{2}}{z^{2}(\tau )}e^{-|t|/\tau }$$

**Figure 5:**
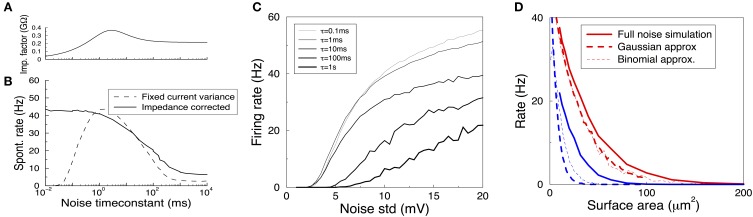
**Spontanous action potentials in the HH model driven with Gaussian noise with varying amplitude and correlation time**. **(A)** The impedance factor *z*(τ) gives the voltage fluctuations resulting from a correlated noise current as a function of the noise correlation time. It is used to ensure the voltage variance is identical as the time constant of the noise is varied. Membrane area of 1000 μm^2^. **(B)** Spontaneous activity as a result of a Gaussian current noise, as a function of the correlation time of the noise. When the variance of the current is fixed, the resulting rates vary strongly depending on the correlation time (dashed curve). However, when the noise is calibrated to yield the same variance in the membrane voltage irrespective of the noise correlation time, the firing rate is much less variable even across six orders of magnitude (solid curve). **(C)** The firing rate vs. the impedance corrected current noise (expressed in σ_*V*_) for various values of the noise time-constant. **(D)** The spontaneous firing rate vs. membrane area for K^+^ (red) and Na^+^ (blue) noise and their various approximations. Solid curve: full simulation, redrawn from Figure 1B. Thick dashed curve: approximation using Gaussian noise with identical variance and time-constant. Thin dashed curve: binomial approximation of the noise. In particular for Na^+^ noise the approximations yield rates that are substantially too low.

The function *z*(τ) is an impedance that relates the voltage variance to the current variance of injected colored noise with time-constant τ. It is given by *z*^2^(τ) = ∫ *S_I_*(*f*)|*Z*(*f*)|^2^*df*, where $S_{I}(f)=\frac{4\tau }{1+{\left(2\pi f\tau \right)}^{2}}$ is the power-spectrum of the noise current, and *Z*(*f*) the linearized impedance of the HH model. Its shape reflects the resonance in the impedance, Figure 5A. As a result of this impedance correction the membrane voltage variance in the limit of small fluctuations equals σ^2^_*V*_ and is thus independent of τ.

When the amplitude of the noise is scaled up, spontaneous spikes appear. The resulting spontaneous rate is shown in Figures 5B (black solid curve),**C**. Remarkably, across six orders of magnitude in the noise time-scale, ranging from noise much faster than any channel or membrane to extremely slow noise, the spontaneous rate varies only one order of magnitude (from 40 to 5 Hz for the noise in Figure 5B). With less noise this ratio can be larger as slow noise might then be unable to evoke the spontaneous spikes, Figure 5C. Furthermore, the relation is monotonic, which is useful when comparing two noise sources. The shape of the curve highlights that fast fluctuating noise is typically an effective driver of neurons, while slow varying noise will tend to inactivate the Na channel close to threshold and is less effective.

Instead, the *current* variance is a much worse predictor of the firing rate (dashed line). Note that in the limit of very slow noise, the dynamics decouple and the spontaneous rate equals ∫ *P*(*I*)*f*(*I*)*dI*, where *P*(*I*) is the distribution of currents and *f*(*I*) is the neuron's deterministic f-I curve.

Finally, we ask if we can use these results to estimate the spontaneous rates caused by K^+^, Na^+^, or in fact any arbitrary channel noise. In the previous section the noise currents were approximated by colored Gaussian noise (an OU process) with variance and correlation time derived from the channel kinetics at the resting voltage, and filtered by the membrane linearized around rest. These approximations hold very well in the subthreshold regime, i.e., for small noise—equivalent to large membrane areas — see Figure 4E, but it is a priori unclear whether they also hold for larger noise amplitudes when spontaneous spikes appear.

We used a colored Gaussian current noise to model the K^+^ and Na^+^ noise, and injected this into a deterministic HH model. For example, in case of the K^+^ channel the variance according to the above sections is

$$\langle \delta I^{2}\rangle =\rho_{K}A{\left[(V_{rest}-E_{K})\gamma_{K}n_{\infty }^{4}(V_{rest})\right]}^{2}$$

and its correlation time is τ_*n*_/4. The spontaneous rate of the neuron driven by this noise was examined as a function of the membrane area. Although this could be accidental, for K^+^ noise, the noise model gave an almost perfect fit to the fully stochastic simulations, Figure 5D compare solid and thick dashed curves. However, the approximated Na noise lead to far too few spontaneous spikes, Figure 5D. Its standard deviation had to be increased by some 50% to fit the simulated spontaneous rates. This need for a fudge factor shows that for smaller areas the Na noise is not well described by additive, colored Gaussian noise. There are many possible cause for this mismatch: the binomial instead of Gaussian current distribution (which additional simulations showed to be a small effect, Figure 5D thin dashed curve), the voltage dependence of the impedance, the dynamics of the full HH system, and likely most important, the strong dependence on the noise current on the membrane voltage, Figure 3E.

In summary then, while we find that sub-threshold noise can be estimated accurately, caution is needed when extrapolating to the spiking regime.

### 3.4. Application to CA1 pyramidal neuron model

Above we showed how to break down the factors determining a given ion channel type's contribution to voltage noise and spontaneous spiking, using the Hodgkin–Huxley squid axon model as an example. However, our approach is completely general and could in principle be applied to any neuron model. To demonstrate its straightforward application, we now perform the same analysis on a well-studied mammalian cell type: the rodent hippocampal CA1 pyramidal neuron. We studied a single-compartment model of this cell type using a well-validated model from the literature (Migliore et al., 1999; Jarsky et al., 2005). The original model contained three active channel types: an Na^+^ channel, an A-type K^+^ channel, Ka, and a delayed rectifier K^+^ channel, Kdr. We built stochastic versions of these active conductances with parameters exactly as previously used (Jarsky et al., 2005).

**Figure 6:**
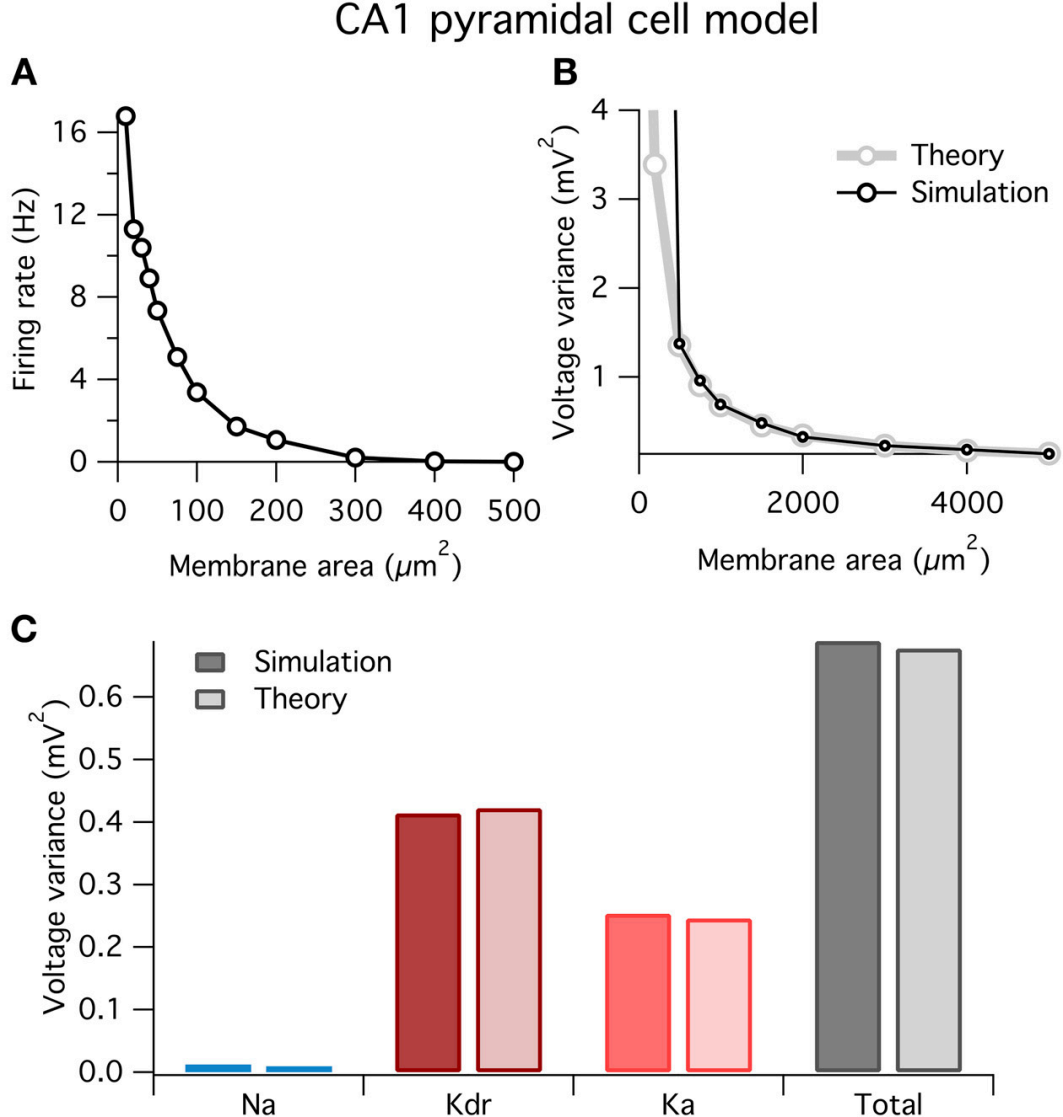
**Spontaneous firing and voltage noise in a stochastic single-compartment model of hippocampal pyramidal neuron**. **(A)** Spontaneous firing rate as function of membrane area for CA1 pyramidal cell model. Qualitative behavior is similar to Hodgkin–Huxley model, Figure 1. **(B)** Comparison of voltage noise variance from theory and simulation as a function of membrane area. As with Hodgkin–Huxley model (Figure 4F), theory is a good predictor for large membrane areas when there is no spontaneous spiking. **(C)** Contributions to voltage variance from the different ion channel types in the model, for both theory and simulation. Two types of potassium channels dominate the noise in this model. Membrane area of 1000 μm^2^.

At small areas the model fired spontaneously (Figure 6A), similar to the HH model above (Figure 1). Also, similar to before, our theory well predicts the variance of voltage fluctuations for large membrane areas, but diverges from the simulation results for small areas <500 μm^2^ when the neuron spikes (Figure 6B).

In Figure 6C we plot the contributions to voltage variance from each of the three voltage-dependent ion channel types in the CA1 pyramidal neuron model. Similar to the HH model above, noise from K^+^ channels dominates over noise from Na^+^ channels. Of the two K^+^ channel types in the model, the delayed rectifier K^+^ channel contributes more voltage noise than the A-type K^+^ channel. Interestingly, Ka has a larger current noise variance than Kdr (not shown), but because Ka has faster kinetics than Kdr, its current noise is more heavily filtered by the membrane capacitance leading to a switch in the relative magnitudes of their contributions to voltage variance.

These results demonstrate two points: first, our method is readily applicable to any neuron model; and second, the dominant source of ion channel noise depends on the physiological details of the neuron. However, it should not be concluded from these results that K^+^ channels will always contribute more noise than Na^+^ channels. Substantial channel noise can arise from channels permeable to any ion: Na^+^, K^+^ or any other. The relative amplitudes and effects of channel noise simply depend on all of the earlier outlined factors and will need to be evaluated on a case-by-case basis.

## Discussion

We have picked apart the various factors that determine a specific channel population's contribution to membrane noise. Although applied only to the Hodgkin Huxley and hippocampal pyramidal neuron models here, the method is applicable to any voltage gated channel model. In summary the factors are:

1. Channel open probability at rest, *p_o_*. The s.d. is proportional to $\sqrt{p_{0}}$, provided *p*_0_ ≪ 1.
2. Number of channels, *N*. The s.d. of the fluctuations in open channel number is proportional to $\sqrt{N}$.
3. Reversal potential. Channels with a larger driving force have a larger single-channel current and hence larger amplitude population current fluctuations.
4. Single channel conductance, γ. The s.d. of current fluctuations is proportional to γ.
5. Channel kinetics. Because the membrane capacitance acts as a low-pass filter, in general the current noise from channels with slower gating kinetics are less attenuated than current noise from channels with faster gating kinetics.

Another qualitative factor is the polarity of current flow. Open Na^+^ channels further depolarize the cell, hence increasing the probability for other Na^+^ channels to open and acting as a positive feedback loop. Hence, stochastic Na^+^ channels increase excitability of the cell through regenerative depolarizing excursions in membrane potential (Dudman and Nolan, 2009). Open K^+^ channels, in contrast, hyperpolarize the cell and act as negative feedback to changes in membrane potential. This negative feedback coupled with their relatively slow kinetics can, in some cases, enable stochastic K^+^channels to drive sub-threshold oscillations (Schneidman et al., 1998).

The combination of these factors yields an accurate prediction of the membrane voltage noise. While it is possible to obtain a coarse estimate of spontaneous firing rates, this is far from perfect and highlights two current hiatus in the theory, namely, the absence of an accurate phenomological model for channel noise when the noise can not be assumed to be small, and the lack of theory for colored noise driven spontaneous activity.

In the case of the stochastic HH model we have shown that the fluctuations from stochastic gating of potassium channels is the dominant source of noise by three different measures. First, a HH model where only K^+^ channels gate stochastically spontaneous fires at higher rates than a HH model where only Na^+^ channels gate stochastically (Figure 1) (Skaugen and Walløe, 1979; Schneidman et al., 1998; van Rossum et al., 2003). Second, examining the dynamics of Na^+^ and K^+^ currents in the milliseconds preceding a spontaneous action potential in the “all stochastic” HH model shows that, on average, spikes are generated by a drop in K^+^ current that precedes the increase in Na^+^ current (Figure 2). Third, direct calculation of the voltage noise spectra from each channel population at resting potential shows that K^+^ channel fluctuations contribute ~ 75% of the total membrane noise (Figure 4). This finding, although consistent with results reported by Schneidman et al. (1998); van Rossum et al. (2003), is in contrast with other simulations (Chow and White, 1996; Faisal et al., 2005). We discuss these two studies separately.

Chow and White (1996) used approximate analytical methods to directly calculate the spontaneous firing rate in the stochastic HH model, and compared the predictions to numerical simulations to find apparently very good agreement. Our own simulations produce quantitatively similar results to their simulations (data not shown), so it is likely that their simulated data are correct. However, their analytical calculations were based on the assumption that spontaneous spiking is driven *solely* by stochastic activation of Na^+^ channels. No matter how elegant, their result can not be accurate as it ignores the K^+^ noise, which is the main cause for spontaneous firing (Figure 1). If anything, their analytical model should be a better approximation of our simulations when K^+^ channels are modeled deterministically. However, their calculations do not match this. The errors could have arisen in any of the multiple approximating steps necessary for their calculation. For example, they assume a static absolute voltage threshold when in reality the HH model has (1) no hard threshold for any type of stimulus (Izhikevich, 2007) and (2) different apparent spike thresholds for stimuli of different temporal structure (Koch, 1999). A more fruitful method for future studies could be to derive a higher-dimensional version of the spike threshold that incorporates both fast and slow channel variable states (Newby et al., 2013).

Faisal et al. (2005) find in cable axon HH models that Na^+^ channels contribute more to spontaneous spiking than K^+^ channels. We believe this to be a numerical simulation error as it is inconsistent with our simulations (when implemented with both PSICS and NEURON), and also those of Schneidman et al. (1998) and van Rossum et al. (2003)—implemented with the “NeuronC” simulator (Smith, 1992). Furthermore, we found the K^+^ channels to be dominant not only in single compartment models but in cable structures as well. Without access to their simulator, it is difficult to tell where the discrepancy lies. Nevertheless, it is possible that Na^+^ channel noise does drive spontaneous spiking in models other than the HH squid giant axon. Our general theory should help to quickly estimate such possibilities without resorting to full simulations.

## Author contributions

Conception: Cian O'Donnell. Carried out analytical and computer calculations, wrote the paper: Cian O'Donnell and Mark C. W. van Rossum.

## Funding

Cian O'Donnell was supported by EPSRC/BBSRC/MRC through the Doctoral Training Centre in Neuroinformatics.

### Conflict of interest statement

The authors declare that the research was conducted in the absence of any commercial or financial relationships that could be construed as a potential conflict of interest.
